# Identifying continence options after stroke (ICONS): a cluster randomised controlled feasibility trial

**DOI:** 10.1186/1745-6215-15-509

**Published:** 2014-12-23

**Authors:** Lois H Thomas, Caroline L Watkins, Christopher J Sutton, Denise Forshaw, Michael J Leathley, Beverley French, Christopher R Burton, Francine Cheater, Brenda Roe, David Britt, Joanne Booth, Elaine McColl, The ICONS Project Team and the ICONS Patient, Public and Carer Involvement Groups

**Affiliations:** School of Health, University of Central Lancashire, Victoria Street, Preston, PR1 2HE UK; School of Healthcare Sciences, Bangor University, Ffriddoedd Road, Bangor, LL57 2EF UK; School of Nursing Sciences, University of East Anglia, Norwich Research Park, Norwich, NR4 7TJ UK; Evidence-Based Practice Research Centre, Edge Hill University, St Helens Road, Norwich Ormskirk, L39 4QP UK; Division of Primary Care, University of Liverpool, Brownlow Street, Liverpool, L69 3GL UK; School of Health, Glasgow Caledonian University, Cowcaddens Road, Glasgow, G4 0BA UK; Newcastle CTU, Institute of Health and Society, Newcastle University, Richardson Road, Newcastle upon Tyne, NE2 4HH UK

**Keywords:** Cluster randomised controlled trial, Feasibility, Stroke, Urinary incontinence

## Abstract

**Background:**

Urinary incontinence (UI) affects half of patients hospitalised after stroke and is often poorly managed. Cochrane systematic reviews have shown some positive impact of conservative interventions (such as bladder training) in reducing UI, but their effectiveness has not been demonstrated with stroke patients.

**Methods:**

We conducted a cluster randomised controlled feasibility trial of a systematic voiding programme (SVP) for the management of UI after stroke. Stroke services were randomised to receive SVP (n = 4), SVP plus supported implementation (SVP+, n = 4), or usual care (UC, n = 4).

Feasibility outcomes were participant recruitment and retention. The main effectiveness outcome was presence or absence of UI at six and 12 weeks post-stroke. Additional effectiveness outcomes included were the effect of the intervention on different types of UI, continence status at discharge, UI severity, functional ability, quality of life, and death.

**Results:**

It was possible to recruit patients (413; 164 SVP, 125 SVP+, and 124 UC) and participant retention was acceptable (85% and 88% at six and 12 weeks, respectively). There was no suggestion of a beneficial effect on the main outcome at six (SVP versus UC: odds ratio (OR) 0.94, 95% CI: 0.46 to 1.94; SVP+ versus UC: OR: 0.62, 95% CI: 0.28 to 1.37) or 12 weeks (SVP versus UC: OR: 1.02, 95% CI: 0.54 to 1.93; SVP+ versus UC: OR: 1.06, 95% CI: 0.54 to 2.09).

No secondary outcomes showed a strong suggestion of clinically meaningful improvement in SVP and/or SVP+ arms relative to UC at six or 12 weeks. However, at 12 weeks both intervention arms had higher estimated odds of continence than UC for patients with urge incontinence.

**Conclusions:**

The trial has met feasibility outcomes of participant recruitment and retention. It was not powered to demonstrate effectiveness, but there is some evidence of a potential reduction in the odds of specific types of incontinence. A full trial should now be considered.

**Trial registration:**

ISRCTN Registry, ISRCTN08609907, date of registration: 7 July 2010.

**Electronic supplementary material:**

The online version of this article (doi:10.1186/1745-6215-15-509) contains supplementary material, which is available to authorized users.

## Background

Urinary incontinence (UI) following stroke is common, affecting around half of stroke survivors in the acute phase [1, 2]. As many as 43.5% and 38% remain incontinent at three months and one year, respectively [3]. In longer term stroke survivors (on average nine years post-stroke), UI prevalence has been reported as 17% [4].

Despite availability of clinical guidelines for the management of UI after stroke [5], national audit data [6] suggest incontinence is often poorly managed. In the latest National Sentinel Stroke Audit [6] only 63% of incontinent patients had a plan for continence management, an increase of just 5% since 2004. Improvements in continence have not kept pace with those in other aspects of stroke care, for example establishing a safe swallow, where the proportion assessed has increased from 63 to 83% over the same period. Research also demonstrates a lack of implementation of continence management practices, even by specialist teams working in recognised stroke units [7, 8].

A lack of guideline implementation may reflect the limited evidence to support clinical guidelines; evidence to underpin the management of UI after stroke is poor, with no robust studies evaluating interventions in secondary care [9]. We have developed an intervention that focusses on conservative strategies shown to have some effect on UI in Cochrane systematic reviews [10–12], but not with stroke patients. It comprises a systematic voiding programme (SVP) including a combined package of bladder training and pelvic floor muscle training, or prompted voiding, together with assessment and review.

In this paper, we report findings from a cluster randomised controlled feasibility trial that aimed to explore rates of recruitment and retention of participants, and to provide preliminary evidence of the clinical effectiveness of the intervention. The trial protocol has been published previously [13].

## Methods

### Study setting and design

The study setting was 12 National Health Service (NHS) stroke services in England and Wales. For the purpose of the trial, a stroke service comprised both acute and rehabilitation stroke units. Stroke units were defined according to the definition provided by the Royal College of Physicians of London for the National Sentinel Stroke Audit [14]. We used an open, cluster randomised controlled trial design with non-blinded outcome assessment; all outcomes (with the exception of death) were self-reported.

### Inclusion criteria

Stroke services:

1. specialist acute and rehabilitation services (either separate or combined units).

Patients:

1. Aged 18 years or over with a diagnosis of stroke based on the World Health Organisation (WHO) criteria [15],
2. UI as defined by the International Continence Society [16] as ‘involuntary loss of urine’ and classified as stress UI (any response other than ‘never’ to the Leicester Urinary Symptom questionnaire [17] (LUSQ) question ‘Do you ever leak when you do any of the following?’), urge UI (the response ‘most of the time’, ‘sometimes’ or ‘occasionally’ to the LUSQ question ‘When you get the urge to pass urine, does any leak before you get to the toilet?’), mixed UI (both stress and urge UI) or ‘functional’ UI, defined as mobility or balance restrictions stopping patients reaching the toilet on time or presence of indwelling catheter in the acute phase of the stroke,
3. Conscious (defined as either ‘alert’ or ‘drowsy’ on the ‘Clinical Status on Admission’ item of the European Stroke Database),
4. Medically stable as judged by the clinical team.

### Exclusion criteria

Stroke services:

1. No access to appropriate excess treatment costs (the difference between the cost to the NHS of providing the new treatment and the cost of standard treatment [18]),
2. Inadequate throughput (defined as less than 150 admissions with stroke per annum),
3. Hub and spoke service model (centralised hyper-acute care followed by rehabilitation provision in several local stroke units) as resources did not allow for SVP implementation in several rehabilitation units in one stroke service.

Patients:

1. Pre-existing long-term catheter,
2. Routine intermittent self-catheterisation prior to stroke,
3. Patients unable to consent for themselves, and for whom a consultee did not agree that the patient would wish to be included.

### Participant recruitment

As the trial was randomised by cluster, informed consent was sought from all participants for collection of process and outcome data only. All eligible patients in stroke services randomised to intervention groups were treated using the SVP, regardless of whether or not they consented to data collection.

All patients admitted to participating stroke units were screened by research nurses within 72 hours of admission using a screening form in line with the inclusion criteria. Nursing staff asked each eligible patient whether their name could be given to the research team. If the patient agreed, a member of the research team visited the patient, explained the project, answered any questions, and provided a participant information leaflet. Patients were given at least 24 hours to consider participation and were visited by a member of the research team after this period. Patients choosing to participate signed the consent form at this stage.

For patients unable to consent for themselves, a person able to advise on the presumed wishes of the patient was approached to act in the role of consultee. This is in line with the recommendations of the Mental Capacity Act (2005; United Kingdom) and in line with the expressed wish of the study Patient, Public, and Carer Involvement Groups that everyone who was eligible should have the opportunity to participate.

The trial was approved by the Bradford Research Ethics Committee (reference number: 10/H1302/60), and by research and development departments in the following Trusts and Health Boards: Betsi Cadwaladr University Health Board (no reference number); Blackpool, Fylde and Wyre Hospitals NHS Foundation Trust (reference number: RD0563); Cambridge Universities Hospital NHS Foundation Trust (reference number: AO92132); Cardiff and Vale University Health Board (reference number: 10/CMC/49); Cwm Taf Health Board (reference number: CT/118/10); East Lancashire Hospitals NHS Trust (reference number: 2010/030); Lancashire Teaching Hospitals NHS Foundation Trust (reference number: 1298); North Cumbria University Hospitals NHS Trust (reference number: 124/10); and University Hospitals of Morecambe Bay NHS Trust (reference number: SFRC 471).

### Sample size and randomisation

The sample size was chosen pragmatically, rather than on the basis of a formal power calculation. Our aim was to balance practicalities and the need for reasonable precision in the estimation of effects to inform the sample size calculation for a full trial.

In order to ensure comparability of trial arms with respect to type of unit, quality of care, and throughput, stroke services were placed into four strata of equal size. These were based, in order of priority, on (1) whether they had separate or combined acute and rehabilitation units at the time (in one site, separate units had combined by the start of recruitment); (2) their average performance on the ‘nine key indicators of stroke care’ in the National Sentinel Stroke Audit Phase II (clinical audit) [6]; (3) the number of stroke patients admitted per year. Services were randomly allocated to usual care (n = 4), intervention (n = 4), and supported implementation (n = 4) arms by the Newcastle Clinical Trials Unit. After allocating stroke services to the strata, the randomisation schedule was generated using block randomisation (block length of three) to allocate one site to each arm within every stratum. The software package STATA (version nine, StataCorp LP, College Station, Texas, USA) was used.

### Trial arms

#### Usual care

In services randomised to usual care, participants received usual continence care which could comprise of: checking for urinary tract infection; checking for overflow incontinence; containment using a variety of products (for example absorbent pads) with regular changes; and some form of toileting schedule.

#### Intervention

In addition to usual care, patients in services randomised to the intervention arm received an SVP comprising assessment, conservative interventions, and review. The assessment phase comprised a three-day bladder diary and a comprehensive continence assessment based on a set of evidence-based criteria [19]. Unit staff were encouraged not to use indwelling urethral catheters unless there was a valid reason. Patients who were cognitively able received bladder training and those with cognitive impairment received prompted voiding. Bladder training included three main components: (1) focused education for patients and carers (including information on the anatomy and physiology of the lower urinary tract, the rationale behind the programme, and strategies to suppress the urge to void, for example distraction and relaxation [20, 21]); (2) individualised voiding regimens designed to restore normal voiding patterns by progressively lengthening the time interval between voids, based on assessment of participants’ normal voiding patterns and self-monitoring; (3) patient-held voiding diary, a cognitive intervention designed to promote self-awareness of voiding habits [22, 23].

While the intention was to combine pelvic floor muscle training with bladder training, implementing pelvic floor muscle training required the support of the physiotherapy team in terms of assessing whether participants were able to exercise their pelvic floor muscles. The physiotherapy teams were reluctant to participate in all units and it was therefore not possible to implement this part of the SVP.

For those patients with cognitive impairments, the programme consisted of elements traditionally classified as prompted voiding. Unlike bladder training, prompted voiding is not designed to affect bladder function, but to avoid or minimise episodes of incontinence [24]. Participants were approached according to individualised schedules (for example, every two hours during waking hours), asked if they were dry or wet, and prompted to use the toilet [25]. Verbal praise was offered for correct reporting of dryness, wetness, and successful toileting.

#### Supported implementation

Services randomised to the supported implementation arm of the trial introduced the SVP using an implementation strategy, facilitation, to assist the process of embedding into practice. Facilitation involves supporting and enabling people to change their practices [26, 27]. It has been used successfully in secondary care settings [26, 27] and is currently the focus of an international trial of ‘technical’ and ‘enabling’ facilitation in a nursing home context [28]. Facilitation involves guiding the group towards accomplishing a goal, helping members identify obstacles that may impede progress and enabling them to identify strategies to overcome them [29]. While there was a focus on goal attainment (defined as the normalisation of the SVP), our approach to facilitation primarily focused on ‘enabling’ rather than ‘doing for’ others [30], with an emphasis on developing and empowering both individuals and teams.

We used both internal and external facilitation. The external facilitators were a senior research fellow in evidence-based practice and a consultant nurse in stroke care; both had expertise in leadership and change management. Each had responsibility for two sites. Their role was to help internal facilitators understand how to bring about change in health professional practice in order to embed the SVP. They also supported internal facilitators by way of encouragement, mentoring, and providing feedback. We employed the expertise of at least one specialist practitioner (staff members who were experts in the field of stroke and incontinence) per stroke service allocated to this arm to serve as internal facilitators. This role was typically adopted by stroke unit nursing managers and involved helping teams work together, providing the necessary information and training, maintaining motivation, and giving feedback and practical help when needed.

### Allocation concealment

Within each stratum, stroke services were not informed of their intervention allocation until all stroke services within that stratum were recruited to take part in the trial. However, when two sites required substitution (one site withdrew due to the pressure of other changes within the stroke service and another site was found to have a throughput of only 120 stroke patients per annum) the rest of the stratum were already aware of their allocation.

### Blinding

Once all stroke services within a stratum were recruited, each was made aware of their allocation, as were staff identifying and recruiting trial participants from within that service. Outcome assessment was by self-reported measures, with data collection supported by research nurses for participants who were still inpatients at six and 12 weeks post-stroke, therefore blinded outcome assessment was not possible. The trial statistician was not blinded during the analysis, although the statistical analysis plan was finalised prior to any outcome data being available.

### Baseline data

Patient characteristics recorded at baseline included: age*; sex; ethnicity; consciousness level on admission (defined as either ‘alert’ or ‘drowsy’ on the ‘Clinical Status on Admission’ item of the European Stroke Database); side of body affected by stroke; type of stroke; stroke sub-type (Oxfordshire Community Stroke Project (OCSP) classification [31]); day seven Barthel Index [32]; pre-stroke Modified Rankin Scale (mRS [33])*; pre-stroke living circumstances*; LUSQ (pre- and post-stroke [17]); type of UI (urge UI, stress UI, mixed UI, ‘functional’ UI, or unclear); cognitive ability (six-item Cognitive Impairment Test [34]); verbal sub-section of the Glasgow Coma Scale [35]*; ability to lift both arms off the bed*; ability to walk independently*; International Consultation on Incontinence Questionnaire (ICIQ-UI Short Form [36]); Incontinence Severity Index (ISI [37]); and the EuroQol (EQ-5D-3 L [38]).

The six factors highlighted with a * above form the Edinburgh stroke case-mix adjuster [39] and were collected to enhance patient-level prognostic adjustment of the statistical modelling of outcomes.

### Outcome data

Outcome data were collected at six and 12 weeks post-stroke via postal questionnaires or questionnaire completion supported by research nurses if participants were still inpatients at either time point. A reminder, with an additional copy of the questionnaire, was sent if postal questionnaires were not received 14 days after sending the original. If no response was received, participants were telephoned and data collected either over the telephone or in an arranged visit where the participant preferred. Those participants recruited to the trial after one of the scheduled outcome assessments were not subjected to outcome data collection for that time point.

### Main outcome

The main effectiveness outcome was participant incontinence (presence or absence) at six weeks as measured by the ICIQ-UI Short Form. Absence of incontinence was defined as the response ‘never’ to question three, ‘How often do you leak urine?’. Presence of incontinence was defined as any other response to question three (ranging from ‘about once a week or less often’ to ‘all the time’). This outcome was also measured at 12 weeks post-stroke.

### Additional outcomes

Additional effectiveness outcomes measured at six and 12 weeks post-stroke were: quality of life measured using the Incontinence Quality of Life Instrument (IQoL [40, 41]) and the EQ-5D-3 L [38]; frequency and severity of incontinence ascertained using the ISI; urinary symptoms measured using the LUSQ, including presence of urge and stress incontinence as defined by Shaw *et al.* [17] respectively as the response ‘most of the time’, ‘sometimes’ or ‘occasionally’ to the LUSQ question ‘When you get the urge to pass urine, does any leak before you get to the toilet?’, and any response other than ‘never’ to the LUSQ question ‘Do you ever leak when you do any of the following?’; activities of daily living (ADLs) measured using the Barthel Index; and death.

A further exploratory effectiveness outcome, continence at discharge, was added to those reported in the protocol. The purpose of this was to capture outcome at the point where participants’ exposure to the intervention ended; it was measured as described above under the Main outcome section.

### Statistical analyses

The primary analysis was performed on the basis of the intention-to-treat principle. Outcome data were collected from all consented patients whenever possible, regardless of their level of subsequent engagement with the allocated intervention programme. For the six-week outcome time point, outcome data received no later than 10 weeks post-stroke were included in the primary analysis; for the 12-week outcome time point, all data received were included with no restriction on when questionnaires were received.

To account for cluster randomisation we used mixed-effects modelling for continuous, ordinal, and dichotomous outcomes to compare the two groups. Baseline measures of the outcome variables (where appropriate), stroke sub-type, and the other prognostic patient-level information (via the Edinburgh case-mix adjuster) were included as individual-level covariates in the models for outcome data.

Missing outcome data were imputed according to the particular outcome. For the primary analysis, dichotomous and ordinal outcomes for those who withdrew, died, or were otherwise lost to follow-up were imputed using a worst-case scenario (for example, for the primary outcome variable, all those for whom incontinence status was not recorded at the respective time points post-stroke were assumed to be incontinent). For continuous outcomes (IQoL), the primary analysis used a non-parametric multiple imputation approach [42]. Missing baseline data were not imputed. Sensitivity analysis included per-protocol analysis, using varying definitions of ‘receipt of sufficient intervention’ and excluding patients with pre-stroke incontinence, or those catheterised for the entirety of their hospitalisation.

## Results

A total of 12 sites commenced recruitment between January 2011 and January 2012. No site dropped out after recruitment began, each recruiting participants either for at least their planned duration of nine or 12 months or until recruitment ceased at all sites on 31 July 2012. Site recruitment periods ranged from seven to 16 months.

A total of 413 patients were recruited into the trial; 124 usual care, 164 intervention and 125 supported implementation (Additional file 1). A total of 6,060 patients were screened for eligibility; of these, 2,675 (44%) had not had a confirmed stroke. The number of non-stroke patients screened was highest in the intervention (1,515 out of 3,078, 49%) and supported implementation (981 out of 1,999, 49%) arms and lowest in usual care (179 out of 983, 18%). There was a large variation across sites, with non-stroke patients screened ranging from 2 (1%) to 448 (79%).

The proportion of stroke patients eligible for recruitment was similar across trial arms (usual care: 155 out of 804, 19%; intervention: 259 out of 1,563, 17%; and supported implementation: 176 out of 1,018, 17%). Of these, 80% (124 out of 155) were recruited into usual care, 63% (164 out of 259) to intervention and 71% (125 out of 176) to supported implementation. The proportion of eligible patients recruited ranged from 50 to 98% across sites.

Baseline data were collected for all patients. The overall response rate at six weeks was 85% (306 out of 362), excluding 34 patients recruited at more than six weeks post-stroke and 17 who had died (usual care: 96 out of 114, 84%; intervention: 122 out of 139, 88%; supported implementation: 88 out of 109, 81%). At 12 weeks, the overall response rate was 88% (330 out of 374), excluding one patient recruited at 12 weeks and 38 who had died (usual care: 98 out of 112, 88%; intervention: 132 out of 148, 89%; supported implementation: 100 out of 114, 88%).

Patient characteristics at baseline are shown in Table 1. Median age was 79 years (interquartile range (IQR): 70.5 to 85) and was similar between arms. Overall, nearly half were male (189, 46%); with slightly more males in the intervention (86, 52%) arm compared with the usual care (51, 41%) and supported implementation (52, 42%) arms. Median day seven Barthel Index was 4 (IQR: 2 to 7) and similar across all arms. The number of patients with no symptoms on the pre-stroke mRS (and therefore no pre-stroke disability) was 139 (34%) overall; the proportion was slightly higher in the usual care (52, 42%) arm compared with intervention (54, 33%) and supported implementation (33, 27%) arms. The median probability of survival free of dependency at six months (measured by the Edinburgh case-mix adjuster [39]) overall was 0.02 (IQR: 0.01 to 0.08) and was similar across all arms.

**Table 1:** **Patient characteristics at baseline (frequency (%) unless stated otherwise)**

Measure	All sites		Usual care		Intervention		Supported implementation	
	n = 413		n = 124		n = 164		n = 125	
Median (IQR) age	79	(70.5-85)	80	(72-86)	77	(68-83)	81	(74-85)
Male	189	46%	51	41%	86	52%	52	42%
Ethnicity:								
White British	397	97%	123	99%	155	96%	119	95%
Other	14	3%	1	1%	7	4%	6	5%
Type of stroke:								
Ischaemic	350	85%	101	81%	143	88%	106	85%
Haemorrhagic infarct	49	12%	17	14%	14	9%	18	15%
Primary intracerebral haemorrhage	12	3%	6	5%	6	4%	0	0%
Oxfordshire Community Stroke Project (OCSP) Classification:								
TACS, total anterior circulation syndrome	185	46%	37	30%	80	51%	68	54%
PACS, partial anterior circulation syndrome	118	29%	54	44%	31	20%	33	26%
LACS, lacunar stroke	88	22%	28	23%	44	28%	16	13%
POCS, posterior circulation syndrome	14	3%	3	2%	3	2%	8	6%
Side of body affected by stroke:								
Left side	207	50%	58	47%	86	53%	63	50%
Right side	176	43%	55	44%	69	43%	52	42%
Both sides	6	1%	2	2%	2	1%	2	2%
Neither side	21	5%	9	7%	4	2%	8	6%
Median (IQR) seven-day Barthel Index	4	(2-7)	5	(2-9)	3	(2-6.3)	5	(3-7.5)
Pre-stroke Modified Rankin Scale:								
No symptoms	139	34%	52	42%	54	33%	33	27%
No significant disability	166	41%	39	31%	72	44%	55	45%
Slight disability	30	7%	4	3%	19	12%	7	6%
Moderate disability	54	13%	21	17%	10	6%	23	19%
Moderately severe disability	18	4%	7	6%	6	4%	5	4%
Severe disability	2	0%	1	1%	1	1%	0	0%
Pre-stroke living type:								
House	324	79%	94	76%	134	84%	96	77%
Flat	47	12%	21	17%	11	7%	15	12%
Sheltered housing	20	5%	4	3%	8	5%	8	6%
Residential home	15	4%	5	4%	4	3%	6	5%
Nursing home	1	0%	0	0%	1	1%	0	0%
Other	1	0%	0	0%	1	1%	0	0%
Pre-stroke living circumstances:								
Alone	162	40%	51	41%	64	40%	47	38%
With partner	194	47%	57	46%	77	48%	60	48%
With other family	37	9%	11	9%	14	9%	12	10%
With other	16	4%	5	4%	5	3%	6	5%
Speech:								
None	47	11%	10	8%	22	14%	15	12%
Incomprehensible	63	15%	19	15%	22	14%	22	18%
Inappropriate	72	18%	25	20%	32	20%	15	12%
Cognitive ability:								
Confused	83	20%	26	21%	22	14%	35	28%
Orientated	146	36%	44	35%	64	40%	38	30%
Median (IQR) Edinburgh case-mix probability of survival free of dependency at six months	0.02	(0.01-0.08)	0.02	(0.01-0.05)	0.02	(0.01-0.11)	0.02	(0.01-0.08)

The majority of patients had middle cerebral artery strokes (total anterior circulation or partial anterior circulation syndrome; 303, 74%), although the proportion of patients with partial anterior circulation syndrome was higher in the usual care (54, 44%) arm compared with intervention (31, 19%) and supported implementation (33, 26%) arms.

### Main effectiveness outcome

Table 2 shows patient outcomes at six weeks post-stroke. An odds ratio (OR) greater than 1 favours the intervention (intervention or supported implementation arms). Overall, only 66 (29%) patients reported being continent, with another 76 (25%) reporting the presence of an indwelling urethral catheter. There was no suggestion of a beneficial effect on outcome of either intervention relative to usual care, with adjusted OR estimates for the dichotomised form of ICIQ question three of 0.94 (95% CI: 0.46 to 1.94) for the intervention arm and 0.62 (95% CI: 0.28 to 1.37) for the supported implementation arm, and for the original ordinal form of the question, 0.83 (95% CI: 0.49 to 1.38) and 0.89 (95% CI: 0.52 to 1.51), respectively.

**Table 2:** **Patient outcomes at six weeks post-stroke**

	Trial arm										
	Usual care (UC)		Intervention (I)		Odds ratio (95% CI) I versus UC ^e^	Supported implementation (SI)		Odds ratio (95% CI) SI versus UC ^e^	All sites		Intracluster Correlation Coefficient (ICC) ^c^
Questionnaires returned	96		122			88			306		
Catheterised (% of returned)	21	22%	37	30%		18	20%		76	25%	
Potential respondents for incontinence measures (% age of returned)	75	78%	85	70%		70	80%		230	75%	
Primary outcome: presence or absence of incontinence (ICIQ-UI Short-Form question three)					0.94 (0.46-1.94)			0.62 (0.28-1.37)			0
Continent^a^	21	28%	29	34%		16	23%		66	29%	
Incontinent^b^	54	72%	56	66%		54	77%		164	71%	
ICIQ-UI Short Form (UC: 75; I: 83; SI: 70; All: 228)^f^					0.83 (0.49-1.38)			0.89 (0.52-1.51)			0
Never	21	30%	29	41%		16	24%		66	31%	
About once a week or less often	8	11%	7	10%		11	16%		26	12%	
Two or three times per week	12	17%	10	14%		8	12%		30	14%	
About once a day	9	13%	11	15%		12	18%		32	15%	
Several times a day	25	35%	25	35%		22	32%		72	34%	
All the time	0	0%	1	1%		1	1%		2	1%	
Secondary outcomes											
Incontinence Severity Index (ISI) score (UC: 75; I: 84; SI: 68; All: 227)^f^					0.87 (0.48-1.57)			0.84 (0.46-1.56)			0
Median (IQR)	4	0-8	4	0-8		4	1-8		4	(0-8)	
None (0)	21	28%	29	35%		16	24%		66	29%	
Slight (1-2)	6	8%	6	7%		7	10%		19	8%	
Moderate (3-4)	13	17%	11	13%		18	26%		42	19%	
Severe (6-8)	35	47%	38	45%		27	40%		100	44%	
Leicester Urinary Symptom Questionnaire											
Frequency of toilet visits during daytime (UC: 71; I: 71; SI: 68; All: 210)^f^					0.43 (0.22-0.84)			0.60 (0.30-1.22)			0.017
At least every 30 minutes	0	0%	0	0%		0	0%		0	0%	
Every hour	0	0%	0	0%		0	0%		0	0%	
Every 90 minutes	2	3%	2	3%		1	1%		5	2%	
Every 2 hours	2	3%	2	3%		10	15%		14	7%	
Less often than every 2 hours	67	94%	67	94%		57	84%		191	91%	
Types of incontinence											
Urge incontinence present (UC: 75; I: 84; SI: 68; All: 227)^f^	52	70%	50	59%	1.35 (0.67-2.71)	48	71%	0.89 (0.41-1.91)	150	66%	0
Stress incontinence present (UC: 71; I: 78; SI: 57; All: 206)^f^	36	51%	32	41%	1.01 (0.42-2.43)	33	58%	0.82 (0.34-1.61)	101	49%	0
EQ-5D-3 L											
Mobility (UC: 96; I: 114; SI: 88; All: 298)^f^					1.27 (0.69-2.34)			1.37 (0.74-2.53)			
No problems	13	14%	10	9%		6	7%		29	10%	
Some problems	51	53%	44	39%		48	55%		143	48%	
Confined to bed	32	33%	60	53%		34	39%		126	42%	
Self-care (UC: 96; I: 114; SI: 88; All: 298)^f^					0.61 (0.34-1.09)			0.75 (0.40-1.39)			0
No problems	13	14%	13	11%		13	15%		39	13%	
Some problems	49	51%	47	41%		36	41%		132	44%	
Unable to wash or dress	34	35%	54	47%		39	44%		127	43%	
Usual activity (UC: 96; I: 114; SI: 88; All: 298)^f^					0.42 (0.22-0.81)			0.79 (0.41-1.51)			0
No problems	6	6%	5	4%		5	6%		16	5%	
Some problems	44	46%	30	26%		34	39%		108	36%	
Unable to perform	46	48%	79	69%		49	56%		174	58%	
Pain or discomfort (UC: 95; I: 111; SI: 84; All: 290)^f^					0.66 (0.38-1.16)			0.63 (0.34-1.14)			0
None	62	65%	58	52%		52	62%		172	59%	
Moderate	32	34%	48	43%		29	35%		109	38%	
Extreme	1	1%	5	5%		3	4%		9	3%	
Anxiety or depression (UC: 95; I: 107; SI: 83; All: 285)^f^					0.56 (0.33-0.95)			0.86 (0.49-1.51)			0
None	51	54%	47	44%		47	57%		145	51%	
Moderate	42	44%	54	50%		33	40%		129	45%	
Extreme	2	2%	6	6%		3	4%		11	4%	
Mean (SD) incontinence Quality of life^d^ (UC: 53; I: 43; SI: 38; All: 134)^f^	75	(56.7-90.9)	78.4	(37.5-89.8)	-10.1 (-29.5-9.3)	74.4	(48.3-92.1)	-10.1 (-28.6-8.3)	76	(51.7-91.2)	0.184
Median (IQR) Barthel Index (UC: 96; I: 117; SI: 88; All: 301)^f^	8.5	(3-14)	7	(3-11)	0.68 (0.43-1.07)	7.5	(4-12)	0.70 (0.43-1.13)	8	(3-12)	0
Dead (UC: 118; I: 145; SI: 114; All: 377)^f^	4	3%	7	5%	1.01 (0.43-2.39)	6	5%	0.72 (0.31-1.71)	17	5%	0.0087

^a^Defined as never on the ICIQ-UI Short Form response question three.

^b^Defined as any non-missing response other than ‘never’ on the ICIQ-UI Short Form.

^c^Any ICC estimate <10^-6^ is presented as 0.

^d^Excluding participants recorded as continent or catheterised.

^e^ORs based on imputed data; values >1 favour I or SI over UC.

^f^Data available for UC, I, SI, and all sites.

At 12 weeks, there was no evidence of better outcome on the ICIQ in either intervention arm (Table 3); OR estimates for the dichotomised form of ICIQ question three were 1.02 (95% CI: 0.54 to 1.93) for the intervention arm and 1.06 (95% CI: 0.54 to 2.09) for the supported implementation arm and, for the original ordinal form of the question, 0.97 (95% CI: 0.58 to 1.61) and 1.22 (95% CI: 0.72 to 2.08), respectively.

**Table 3:** **Patient outcomes at 12 weeks post-stroke**

	Trial arm										
	Usual care (UC)		Intervention (I)		Odds ratio (OR) (95% CI) I versus UC ^e^	Supported implementation		OR (95% CI) SI versus UC ^e^	All sites		Intracluster Correlation Coefficient (ICC) ^c^
Questionnaires returned	98		132			100			330		
Catheterised (% of returned)	18	18%	27	20%		13	13%		58	17%	
Potential respondents for incontinence measures (% age of returned)	80	82%	105	80%		86	86%		271	82%	
Primary outcome: presence or absence of incontinence (ICIQ-UI Short-Form question three)											
Continent^a^	24	30%	43	41%	1.02 (0.54-1.93)	27	31%	1.06 (0.54-2.09)	94	35%	0
Incontinent^b^	56	70%	62	59%		59	68%		177	65%	
ICIQ-UI Short Form (UC: 80; I: 104; SI: 86; All: 270)^f^					0.97 (0.58-1.61)			1.22 (0.72-2.08)			0
Never	24	30%	43	41%		27	31%		94	35%	
About once a week or less often	12	15%	9	9%		10	12%		31	12%	
Two or three times per week	12	15%	13	12%		11	13%		36	13%	
About once a day	12	15%	6	6%		10	12%		28	10%	
Several times a day	18	23%	25	24%		27	31%		70	26%	
All the time	2	3%	8	8%		1	1%		11	4%	
Secondary outcomes											
Incontinence Severity Index (ISI) score (UC: 80; I: 102; SI: 86; All: 268)^f^					0.86 (0.50-1.50)			0.92 (0.52-1.64)			0
Median (IQR)	3	(0-6)	2.5	(0-8)		4	(0-8)		3	(0-8)	
None (0)	24	30%	43	42%		28	33%		95	35%	
Slight (1-2)	10	13%	8	8%		6	7%		24	9%	
Moderate (3-4)	19	24%	12	12%		17	20%		48	18%	
Severe (6-8)	27	34%	39	38%		35	41%		101	38%	
Leicester Urinary Symptom Questionnaire											
Frequency of toilet visits during daytime (UC: 73; I: 88; SI: 71; All: 232)^f^					0.85 (0.47-1.54)			1.09 (0.60-1.96)			0.0075
At least every 30 minutes	0	0%	0	0%		0	0%		0	0%	
Every hour	0	0%	0	0%		1	1%		1	0%	
Every 90 minutes	5	7%	2	2%		4	6%		11	5%	
Every 2 hours	10	14%	11	13%		6	8%		27	12%	
Less often than every 2 hours	58	79%	75	85%		60	85%		193	83%	
Type of incontinence											
Urge incontinence present (UC: 79; I: 103; SI: 85; All: 267)^f^	53	67.1%	52	50.5%	1.58 (0.83-2.99)	49	57.6%	1.73 (0.88-3.43)	154	57.7%	0
Stress incontinence present (UC: 72; I: 83; SI: 72; All: 227)^f^	38	52.8%	29	34.9	1.04 (0.45-1.82)	31	43.1	1.82 (0.82-4.01)	98	43.2%	0
EQ-5D-3 L											
Mobility (UC: 96; I: 129; SI: 92; All: 317)^f^					0.92 (0.52-1.62)			0.79 (0.44-1.41)			0
No problems	19	20%	16	12%		10	11%		45	14%	
Some problems	53	55%	62	48%		57	62%		172	54%	
Confined to bed	24	25%	51	40%		25	27%		100	32%	
Self-care (UC: 97; I: 126; SI: 92; All: 315)^f^					0.45 (0.26-0.79)			0.65 (0.36-1.16)			0
No problems	25	26%	21	17%		18	20%		64	20%	
Some problems	42	43%	47	37%		40	43%		129	41%	
Unable to wash or dress	30	31%	58	46%		34	37%		122	39%	
Usual activities (UC: 97; I: 126; SI: 91; All: 314)^f^					0.49 (0.27-0.90)			0.63 (0.34-1.17)			0
No problems	10	10%	9	7%		8	9%		27	9%	
Some problems	43	44%	39	31%		32	35%		114	36%	
Unable to perform	44	45%	78	62%		51	56%		173	55%	
Pain or discomfort (UC: 95; I: 123; SI: 93; All: 311)^f^					0.73 (0.43-1.23)			0.88 (0.50-1.54)			0
None	55	58%	51	41%		50	54%		156	50%	
Moderate	37	39%	58	47%		34	37%		129	41%	
Extreme	3	3%	14	11%		9	10%		26	8%	
Anxiety or depression (UC: 95; I: 122; SI: 92; All: 309)^f^					0.67 (0.39-1.13)			0.95 (0.54-1.67)			0
None	53	56%	47	39%		47	51%		147	48%	
Moderate	37	39%	66	54%		37	40%		140	45%	
Extreme	5	5%	9	7%		8	9%		22	7%	
Mean (SD) Incontinence Quality of Life^d^ (UC: 51; I: 47; SI: 35; All: 133)^f^	72.6	(58.3-83.0)	76.1	(42.5-94.3)	-5.5 (-24.1-13.1)	67.1	(51.1-85.2)	-1.9 (-21.2-17.4)	72.6	(52.9-87.5)	0.216
Median (IQR) Barthel Index (UC: 94; I: 128; SI: 95; All: 317)^f^	11	(4-16)	8	(4-13)	0.71 (0.46-1.11)	11	(6-15)	0.97 (0.61-1.54)	10	(4-15)	0
Dead (UC: 123; I: 159; SI: 122; All: 404)^f^	12	10%	16	10%	1.04 (0.56-1.92)	10	8%	1.15 (0.60-2.19)	38	9%	0

^a^Defined as never on the ICIQ-UI Short Form response question three.

^b^Defined as any non-missing response other than ‘never’ on the ICIQ-UI Short Form.

^c^Any ICC estimate <10^-6^ is presented as 0.

^d^Excluding participants recorded as continent or catheterised.

^e^ORs based on imputed data; values >1 favour I or SI over UC.

^f^Data available for UC, I, SI, and All Sites.

### Other effectiveness outcomes

There was no suggestion of a clinically meaningful improvement in the intervention and/or supported implementation arms relative to usual care at six or 12 weeks post-stroke for any of the other outcomes. At six weeks, 44% (100) of the non-catheterised respondents reported severe incontinence on the ISI, with similar percentages with severe incontinence in each trial arm (usual care: 35, 47%; intervention: 38, 45%; supported implementation: 27, 40%).

At 12 weeks, both intervention arms had a higher estimated odds of continence than usual care with respect to urge incontinence (intervention: OR: 1.58, 95% CI: 0.83 to 2.99, supported implementation: OR: 1.73, 95% CI: 0.88 to 3.43). There was a greater estimated odds of continence with respect to stress incontinence in the supported implementation (OR: 1.82, 95% CI: 0.82 to 4.01) arm but not the intervention (OR: 1.04, 95% CI: 0.45 to 1.82) arm, compared with usual care.

There was a consistent pattern of worse estimated effects of the intervention on EQ-5D-3 L outcomes, but only the confidence intervals for the effect of the intervention arm (relative to usual care) on usual activity and anxiety and depression at six weeks, and on self-care and usual activities at 12 weeks, suggested ORs below one (poorer quality of life).

Overall, 161 (39.7%) participants were continent at discharge; 38 (31%) in usual care, 72 (44%) in the intervention arm, and 51 (41%) in the supported implementation arm. Relative to usual care, the intervention arm had an OR of 1.47 (95% CI: 0.81 to 2.67) of being discharged continent, with supported implementation having an OR of 1.54 (95% CI: 0.83 to 2.85). On combining the two intervention arms, their participants had 1.50 (95% CI: 0.88 to 2.57) times the odds of continence at discharge than usual care.

### Sensitivity analysis

At both six and 12 weeks, findings were insensitive to the exclusion of those catheterised throughout their hospital stay (and also to the exclusion of those who were never incontinent following the removal of a catheter). However, at both time points, odds ratios reduced when those with pre-stroke incontinence were excluded.

## Discussion

### Recruitment

The number of potential participants available for recruitment was affected by the large proportion of people admitted who did not have a stroke, which comprised nearly half of those admitted to the intervention (1,515, 49%) and supported implementation (981, 49%) units. A potential explanation may be hospital policies on bed usage: two intervention units also admitted medical patients when there was a shortage of medical beds in the hospital, while in two supported implementation units only a proportion of beds were designated for patients with stroke (18 out of 26 and 12 out of 28). In one unit, two-weekly rotations of medical staff across geriatric medicine meant a definitive diagnosis of stroke was often delayed; staff seemed reluctant to approach potential participants without this leading to patients being discharged without being approached. Other units may have had ineffective procedures for diagnosing confirmed cases in the emergency department, and/or a lack of nurse specialist and/or stroke-specific consultant physician roles.

Recruitment of patients to the usual care arm was affected by two further factors: throughput of patients and length of the recruitment period. No large hospital (over 600 admissions with stroke per annum) was randomised to this arm. Furthermore, in two hospitals delays in obtaining approvals meant recruitment started much later than planned, but still had to be stopped at the end of July 2012 to allow sufficient time for data analysis and reporting.

The actual numbers of people admitted with a confirmed stroke at individual centres were up to 65% lower per annum than initial figures provided by the Trusts or Health Boards. This difference was particularly marked in the two intervention arms, with overall proportions 33% and 34% lower than estimates provided by the units in the intervention and supported implementation arms, respectively, compared with just 4% lower in usual care. The mismatch between reported and actual numbers of stroke patients meant there were fewer than expected eligible patients. These lower numbers need to be accounted for in our future trial, and in stroke trials more generally.

The proportion of people admitted with stroke that were eligible for the trial (19% in usual care, and 17% in each of the implementation and supported implementation units) was half that expected based on prevalence reported in previous studies [3, 43]. Possible explanations are a larger than expected proportion of patients who were not deemed to be medically stable, continent by the time they were classed as medically stable, or receiving end of life care.

Interpretation of the inclusion criterion ‘medically stable’ was variable across sites and contributed to delays in recruiting patients who were six or more weeks post-stroke (34 patients) and, in one case, 12 weeks post-stroke. Patients tended to be recruited slightly earlier in the usual care arm (mean of 15.6 days post-stroke compared with 21.1 days in the intervention arm and 17.9 in the supported implementation arm). Delays in recruitment could explain the lower than expected number of people with mild incontinence, which could have resolved by the time people were deemed to be medically stable. In intervention arms, delays also affected when patients began the SVP, contrary to current guidance suggesting all those who are incontinent two weeks after diagnosis should be reviewed and a treatment plan developed [5].

A larger percentage had less severe strokes (partial anterior circulation syndrome (PACS)) compared with patients in the intervention arms (usual care: 44%, intervention: 20%, and supported implementation: 26%). While levels of functioning were similar across trial arms at baseline, the greater proportion of patients with PACS may have contributed to better prognosis, both in terms of general recovery and recovery of continence in this trial arm.

In order to minimise the likelihood of baseline imbalances in the full trial, consideration should be given to recruiting patients within 72 hours of admission to the stroke unit. Revised strategies for identifying patients with mild incontinence (for example, including a review of continence within the assessment process on admission to the unit) are also required. In intervention units, the assessment phase of the SVP can begin during this time, with components requiring more active input from patients (for example, bladder training) beginning when patients are deemed by the multidisciplinary team to be ready to start active rehabilitation.

Sensitivity analysis indicates that odds of continence may be reduced when those with pre-stroke UI are excluded. This suggests those with pre-stroke incontinence are at least as, or more likely to benefit from the intervention than those continent pre-stroke; this group of patients should therefore be included in the full trial.

### Participant retention

The proportion of participants for whom outcome data were available at six (85%) and 12 (88%) weeks was acceptable [44] as there was less than 20% attrition. This was a particular achievement given the nature of the patient population: the median Barthel Index score was eight at six weeks and 10 at 12 weeks, indicating severe disability.

### Preliminary evidence of effectiveness

There was no suggestion of a beneficial effect of the SVP (with or without supported implementation) on outcome at six weeks post-stroke. However, almost 50% of intervention-arm patients had received less than two weeks of their allocated intervention by this time point, with around 25% having spent less than seven days on the programme. Outcome findings were similar at 12 weeks post-stroke, although both intervention arms had a higher estimated odds of continence at discharge and an increase in the estimated odds of continence for patients with urge incontinence than usual care; there was a similar increase in the estimated odds of continence for stress incontinence in the supported implementation arm.

While none of these differences were statistically significant, they suggest participants in intervention units were more likely to be continent at discharge, and also point to a potential reduction in the odds of specific types of incontinence, with evidence more consistent across the arms for urge incontinence. This may be explained by our inability to introduce pelvic floor muscle training, with the consequence that the intervention did not specifically target stress incontinence.

Despite extensive liaison with therapy staff, no intervention sites included pelvic floor muscle training as part of a combined intervention with bladder training. Lack of therapist involvement in identifying, assessing, and managing UI after stroke is at odds with the key role recommended by both evidence [45–47] and policy [48]. Delivering the intervention as intended (as a combined intervention of bladder training and pelvic floor muscle training) may have led to a greater improvement in participant outcomes.

Delays in both recruiting patients and beginning the SVP may have attenuated the estimate of preliminary effectiveness at six weeks, particularly given that outcome time points were measured from date of stroke rather than either date of recruitment or date of commencement of the SVP. This feasibility trial has indicated that measuring outcome at 12 weeks may provide a more accurate reflection of preliminary effectiveness, as by this time patients had been on the SVP for a mean of 28.12 (SD: 18.45) days in the intervention arm and 26.36 (SD: 23.33) days in the supported implementation arm. The 12-week time point will be used as the main outcome time point in the full trial. This will be measured from date of consent in order to reduce the time from stroke to consent found in this feasibility trial.

As the majority of patients were discharged before the 12-week data collection point (usual care: 110, 88.7%; intervention: 131, 79.7%; supported implementation: 94, 76.4%), measuring presence or absence of continence at discharge was useful in assessing preliminary evidence of effectiveness as it allowed assessment at the end of the intervention period (the point where effectiveness was most likely to be demonstrated). The extent to which the SVP was continued either at home or in other care environments is unknown, but there is some evidence to suggest patients’ continence status regressed following discharge. Overall, two fifths of patients continent at discharge were incontinent at 12 weeks (22 out of 54, 40.7%); a smaller proportion of patients were incontinent in the intervention arm (7, 29.2%) compared with usual care (8, 57.1%) and supported implementation (7, 43.8%).

The SVP protocol recommended avoiding catheterisation (except for the management of urinary retention or where fluid balance was critical) and reviewing and removing catheters as soon as possible after stroke, in line with current guidance [5]. Nearly half of the patients in intervention arms were catheterised in the acute stage (139 out of 289, 48.1%). While this is much higher than the 20% reported in the 2010 National Sentinel Audit [6], this percentage is of those recruited and cannot necessarily be extrapolated to all people admitted to the stroke unit.

Given the high rate of catheterisation found in this study, more detailed guidance needs to be added to intervention protocols in the future trial, particularly around avoiding unnecessary catheterisation, conducting a ‘trial without catheter’ as soon as possible, and reviewing catheterised patients on a weekly basis.

As the SVP was delivered only in the stroke unit, it is possible patients did not receive the programme long enough to benefit, with intervention and supported implementation arm patients spending an average of 28 days and 37 days receiving the intervention, respectively. The duration in previous studies was typically more than six weeks for bladder training [12], and between 20 days and 32 weeks for prompted voiding [10]. Encouraging staff to begin the SVP as soon as patients are able to begin rehabilitation, and extending the SVP beyond discharge from the stroke unit, should be considered in order to maximise the possibility of a treatment effect.

## Conclusions

This feasibility trial demonstrated that it was possible to recruit and retain participants, although this took longer than planned. There is some evidence of more favourable continence outcomes in intervention arms at 12 weeks, particularly in those participants with urge and (to a lesser extent) stress incontinence, and patients in the intervention arms were more likely to be continent at discharge.

In terms of participant recruitment, the inclusion criterion ‘medically stable’ should be removed, and the recruitment process should begin within 72 hours of admission to the stroke unit to help minimise imbalances at baseline, with progression onto the SVP once patients are judged ready to begin rehabilitative activities.

## Electronic supplementary material

Additional file 1: **Flowchart showing cluster and participant recruitment.** (DOCX 53 KB)

## Abbreviations

ADLs: Activities of daily living
CI: Confidence interval
EQ-5D-3 L: EuroQol quality of life measure
ICIQ-UI Short Form: International Consultation On Incontinence Questionnaire-Short Form
ICONS: Identifying Continence Options after Stroke study
IDMC: Independent Data Monitoring Committee
I-QoL: Incontinence Quality of Life Instrument
ISI: Incontinence Severity Index
LUSQ: Leicester Urinary Symptom Questionnaire
mRS: Modified Rankin Scale
NHS: National Health Service
NIHR: National Institute for Health Research
OCSP: Oxford Community Stroke Project
OR: Odds ratio
SVP: Systematic voiding programme
TMG: Trial Management Group
TSC: Trial Steering Committee
UI: Urinary incontinence
UK: United Kingdom
WHO: World Health Organization.
