# Supplementary material for: Identifying continence options after stroke (ICONS): a cluster randomised controlled feasibility trial
Source: Trials. 2014 Dec 23;15:509. doi: 10.1186/1745-6215-15-509 (PMC4307223; doi:10.1186/1745-6215-15-509)
Supplement: Supplementary file 1 — Additional file 1: Flowchart showing cluster and participant recruitment. (DOCX 53 KB) [file 13063_2014_2381_MOESM1_ESM.docx]

**6**

**wk**

**12**

**wk**
